# A phenomenological analysis of the emotional experiences of graduate preservice student teachers in Indonesia

**DOI:** 10.12688/f1000research.132112.1

**Published:** 2023-05-16

**Authors:** Sunardi Sunardi, Famala Eka Sanhadi Rahayu, Desy Rusmawaty, Istanti Hermagustiana, Dyah Sunggingwati

**Affiliations:** 1 English Language Department of Teacher Training and Education Faculty, Universitas Mulawarman, Samarinda, East Kalimantan, 75123, Indonesia; 2 English Literature of Cultural Science Faculty, Universitas Mulawarman, Samarinda, East Kalimantan, 75123, Indonesia

**Keywords:** emotional geography, student teachers, teaching practicum, teacher education

## Abstract

**Background**: Addressing preservice teachers' emotions can help them develop the emotional connections necessary for successful teaching practicums and professional learning experiences. To understand preservice teachers’ emotions towards their surroundings, the researchers used an emotional geography framework to map out the source of their negative and positive emotions and how those feelings affect their beliefs, teaching style, and paradigms. Emotional geographies were divided into two categories (i.e., emotional distance and emotional closeness), which were further mapped into five major themes (i.e., sociocultural, moral, professional, physical, and political).

**Methods**: This study examined emotional geographies of 15 graduate preservice student teachers of Teacher Training and Education Faculty of Mulawarman University during their teaching practicums. Moreover, this study used a qualitative design involving semi-structured interviews with 15 graduate students who completed their teaching practicums.

**Results**: The participants experienced all emotional geographies (sociocultural, moral, professional, physical, and political) that reshaped their beliefs and teaching styles, especially related to teaching undergraduate students. These emotions are generally experienced when the participants deal with the students and cooperating teachers.

**Conclusions**: It was found that teachers needed to be strict when instilling discipline in their students, develop friendships with their students to maintain classroom control, and require comprehensive preparation to create teacher's confidence and answer their students' questions. In effect, the theoretical framework of emotional geography when teaching undergraduates during practicums gave student teachers valuable experience to cope with all common teaching challenges and professional growth.

## Introduction

Teacher education can mold the personalities of a nation’s future generations. Therefore, high-quality teacher education aims to develop qualified, competent teachers in order to shape a country’s education future, which requires relevant teacher education courses that build and strengthen teacher competencies. One such program is a practicum, which is a required teacher education component from which preservice teachers can acquire professional growth and apply theoretical learning to practical situations ( Hascher & Hagenauer, 2016). Preservice teachers refer to teaching candidates enrolled in post-secondary education-related degree programs who are receiving their initial training before practicing and have not yet taught in a classroom ( Milner *et al*., 2003). In Indonesia, preservice teacher practicums are defined under the Ministry of Research, Technology, and Higher Education Regulation No. 55 of 2017 on Teacher Education Standards, paragraph 9, which states that all teacher training institutions must provide practicum programs that involve microteaching and school-based practicums.

Professional experience and/or teaching practicums are bridges between theory and practice and provide authentic preservice experiences for developing pedagogical skills and a familiarity with the teaching education landscape ( Kaldi & Xafakos, 2017), which is critical because good teaching performances are related to strong pedagogical foundations ( Hargreaves & Shirley, 2012). Therefore, teaching practicums provide student teachers with experience in teaching in terms of teacher education and preparation programs. Studies into preservice or student teachers can be valuable because such teachers have dual roles as both students and teachers, and their success in teacher education programs may reveal their potential as future educators ( Lipka & Brinthaupt, 1999). Several studies have been conducted on preservice teachers, which involved assessing their knowledge and competence ( Alkharusi *et al*., 2011; Petrovici & Masari, 2014; Ragawanti, 2015; Valli *et al*., 2013; Walton & Rusznyak, 2013), attitudes toward teacher programs and the teaching profession ( Bhargava and Pathy, 2014; Mule, 2006), the challenges ( Karunagaran & Saimin, 2019; Shah *et al*., 2021), anxiety ( Agustiana, 2014; Daud *et al*., 2019), and perceptions ( Adodo, 2013; Kumazawa, 2013; Sun & van Es, 2015; Thomas & O’Bannon, 2013; Zhu, 2017). While much of this study emphasizes the need to improve teacher education quality and preservice teacher abilities, few studies were noted to examine preservice teachers’ emotions during their practicums.

Emotional effects have frequently been ignored because of a lack of awareness of the effects of teacher emotions on teaching quality. However, understanding teachers’ feelings could provide a more comprehensive perspective on teacher education effectiveness. Therefore, this study investigated the feelings that preservice teachers had during their teaching practicums and the influence of these emotions had on their teaching paradigms, beliefs, and teaching styles. One of the tools to assess teachers’ emotional geography in the classroom is by examining their emotion through Hargreave's emotional geography framework. Emotional geographies describe the patterns of closeness and distance in human interactions that shape the emotions we experience about relationships to ourselves, each other, and the world around us ( Hargreaves, 2001b). Hargreaves (2001b) claimed that understanding teachers’ emotions is crucial because it can influence the classroom environment and the conduct of the learning activities.

Hargreaves’ (2001b) emotional geography framework can also be used to map a teacher’s series of feelings. Hargreaves (2001b) defined five emotional geographies (i.e., sociocultural, moral, professional, physical, and political) as geographical and experiential patterns of closeness/distance in human connections that form, configure, and color feelings about ourselves, the world, and one another. Various studies revealed that teachers encountered emotional geographies in their interactions with parents ( Chen & Wang 2011; Sanhadi Rahayu & Asanti 2021), colleagues ( Hargreaves, 2001a), students ( Hargreaves, 2000), and principals ( Lassila *et al*., 2017) as well as during the teaching process itself ( Hargreaves, 1998a; Hargreaves & Tucker, 1991). However, the investigation of emotional geography of preservice teacher during the practicum has remained unseen by many scholars. Therefore, the present researchers want to fill this gap through this study.

As aforementioned, a teaching practicum provides real-life experience in teaching students in the classroom. However, preservice teachers encounter various obstacles and experience a variety of emotions during their teaching practice. They have to deal with various settings involving students and the sociocultural contexts in the institution in which they are teaching ( Riesky, 2013). This study revealed the difficulties preservice teachers experienced due to their emotional beliefs ( Riesky, 2013). Dotger *et al*. (2011) simulated parent–teacher and preservice teacher conferences to examine preservice teachers’ emotions, with the findings suggesting that their emotional geography could be classified into three distinct frameworks: professional, moral, and political. These emotional geographies revealed that preservice teachers were most apprehensive when engaging with parents about their children or the school, especially when the parents exhibited various emotions (e.g., support, concern, frustration, and fury). Rejeki Kristina, and Drajati (2018) profiled a female English instructor who taught in a rural location in Indonesia and developed an emotional geography sociocultural framework that depicted the participants’ feelings and discomfort about their cultural and social background and the rural environment in which they had been placed. Liu (2016) documented the experiences of an immigrant teacher in a professional community and how they overcame their emotional barriers to community understanding. These studies suggest that diverse emotional, physical, moral, sociocultural, professional, and political geographies shaped the teachers’ positive and negative feelings. Although numerous studies have examined the emotional experiences of preservice teachers during their teaching practicums ( Dotger *et al*., 2011; Karunagaran & Saimin, 2019; Riesky, 2013; Thomas & O’Bannon, 2013), little attention has been given to their emotional geography as they interact with the students, their cooperating teachers, and other faculty members during their initial teacher education. Therefore, this study aims to investigate graduate preservice student teachers’ emotional geographies during their teaching practicums through Hargrave's emotional geography framework. Addressing preservice teachers' emotions has the potential to strengthen emotional bonds between preservice student teachers, cooperating teachers, and students, allowing them to develop the emotional connections required for fruitful teaching practicums and professional learning experiences during their initial teacher education.

## Literature review

### Emotional geographies in teaching

A teacher’s emotions are indicators that can be used to explore aspects beyond the teacher’s professional relationships ( Hargreaves, 2001a). Teaching is not only a cognitive and behavioral practice where elaborating teaching depends only on what they know and what they should do in the class. Instead, teaching involves another factor, such as their feeling when teaching, which can be a help or a threat for their professionalism.

A teacher’s experience of teaching can be affected by their relationships with the students ( Hargreaves, 2000), parents ( Hargreaves & Lasky, 2004), their colleagues ( Hargreaves, 2001a), principals, and educational changes ( Hargreaves, 1998a, 1998b), with the feelings from these relationships affecting teaching styles, judgments, and the conduct of the class learning activities. Therefore, understanding these positive and negative emotions related to teaching, could reveal the underlying decision-making and problem-solving contexts in the classroom.

Understanding teacher emotions is important for teachers with extensive teaching experience and student teachers during their teaching practicum. Teaching practicums allow preservice teachers to experience a real class and apply the knowledge they have learned. Following teaching plans may not be as easy as one believes and may evoke positive and negative emotions depending on how the threats in the class are perceived. These stressful situations guide the problems that need to be overcome, and the feelings being experienced could lead to changes in preservice teachers’ perceptions, beliefs, and teaching styles ( Yuan & Lee, 2014).

### Graduate preservice student teachers

Graduate preservice teachers (GPSTs) are graduate students enrolled in master’s degree programs who are required to complete their teaching practicums before graduation ( Geng *et al*., 2016). Although the term ‘preservice teachers’ is widely used, the preservice teachers in this study were slightly different because they were studying for their master’s degrees rather than bachelor’s degrees. Graduate English education program students are required to complete a teaching practicum comprising three to five classes with undergraduate students in similar majors and to teach the classes similarly to the undergraduate student lecturers. These teaching practicums provide GPSTs with additional teaching experience by allowing them to teach undergraduate students whose characteristics differ distinctly from the secondary school students they taught as part of their bachelor’s degrees, thus increasing their knowledge about dealing with adult students and positioning them for possible future employment as lecturers ( Sunardi, 2018).

### Teaching emotions

A classroom is an emotional place. The students spend endless hours attending class, finishing projects, taking tests, and building social relationships contributing to life goals—holding a degree in education today has been more personal, social, or financially significant. Emotions like curiosity, interest, hope, pride, rage, anxiety, shame, confusion, irritation, and tedium are common, prevalent, and intense in these contexts ( Pekrun & Linnenbrink-Garcia, 2015; Zhang & Zhu, 2008).

The significance of emotions in education extends to teachers, principals, and administrators in equal measure. For instance, teachers are responsible not only for imparting knowledge but also for instilling a passion for the subject and enthusiasm for learning ( Pekrun & Linnenbrink-Garcia, 2015). Student teachers will learn emotions early in the teaching process, in this case, during their teaching practicum. Teaching practicum can project the real teaching atmosphere and give the student teacher valuable experiences to expose themselves to emotions in teaching. Typically, these feelings emerge when teachers' objectives, standards, and beliefs interact with those of other classroom participants during routine school activities ( Hargreaves, 1998a). Thus, teachers experience various emotions, from happiness and pleasure when a lesson proceeds as intended to frustration and anger when working with difficult administrators ( Schutz, 2014; Sutton & Wheatley, 2003). If they successfully generate enthusiasm for the course material, the motivational effects should extend well beyond the course. However, failing to maintain good teaching by standards will lead them to feel anxiety or boredom. ( Pekrun & Linnenbrink-Garcia, 2015; Sutton & Harper, 2009; Titsworth *et al*., 2010)

## Methods

### Ethics

This study was approved by Universitas Mulawarman and Ethical Clearance Committee (Protocol Number: 061/UN17.5/PP/2020). Written informed consent was obtained from all participants.

### Participants

This study investigated the emotional geographies of Graduate Pre-Service Student Teachers (GPSTs) majoring in English Education at Mulawarman University, who had finished their teaching practicum in 2019. There were 21 students who were first recruited and contacted for taking part in this study online via Zoom, but only 15 students were able to proceed with the interview. Six other students could not participate in this study due to limited access to the internet in their hometown as most of the students had returned home because of COVID-19 restrictions ( Ministry of Education and Culture of Indonesia, 2020). Participant profiles are shown in Table 1.

**Table 1.** GPST profile.

No.	Participant	Teaching experience	Current job
1	Participant 1	6 months	Substitute junior high school teacher
2	Participant 2	>15 years	Senior high school teacher and university lecturer
3	Participant 3	2 years	English course instructor
4	Participant 4	2 years	Senior high school teacher
5	Participant 5	2–3 years	English course instructor
6	Participant 6	6 months	Housewife
7	Participant 7	>2 years	Junior high school teacher and university tutor
8	Participant 8	3 years	General affairs officer
9	Participant 9	<6 months	French tutor
10	Participant 10	<6 months	Recent graduate
11	Participant 11	1 year	Not working
12	Participant 12	6 years	Junior and senior high school teacher
13	Participant 13	3 years	Junior high school teacher
14	Participant 14	<6 months	Entrepreneur
15	Participant 15	3 years	Junior and senior high school teacher

All participants were enrolled in the TESOL teacher training program and had various previous education and teaching experiences. Most participants had been teaching for several years, both in formal education (e.g., teaching at school) and informal education (e.g., teaching in an English course). In contrast, three participants were relatively new to teaching, especially teaching university students.

### Research procedure

This research was conducted from March to April 2020. Because of COVID-19 restrictions ( Ministry of Education and Culture of Indonesia, 2020), the thirty to sixty minute semi-structured interviews were conducted online via Zoom; with two interviews conducted for each participant. Then, the participants were contacted by the researcher (S) to ask for their willingness and availability to participate in the study. The researcher (S) took the participants’ contact information from the postgraduate program of English Education and he also provided the link for the Zoom meeting. There were 15 students who were able and available to attend an interview with the researcher (S). The first interview was exploring their emotional geographies and in the second interview, researchers verified the interview transcriptions with the participants to ensure their answers were recorded correctly. In both interviews, the participants voluntarily gave consent to the use of their experience as data for this research. The transcript was double-checked by the participant to ensure the result were accurate.

### Data analysis technique

A phenomenological approach was adopted for data analysis and included three steps: coding the emotions, identifying and/or interpreting the causes of the reported emotions, and identifying the themes based on the emotional geography framework of Hargreaves (1998a). In the first *coding emotion* step, the interview transcripts were searched for passages that included information on teachers’ emotions, to which an emotional geography coding scheme was applied. The emotions (e.g., annoyance, happiness, and joy) were plotted as either *positive* or *negative* and coded accordingly. The second step in the analysis identified and/or inferred the cause of the participants’ reported emotions as reported by participants, the descriptions for which were paraphrased and described using qualitative content analysis. Statements describing similar causes were merged and a summary of the inferred causes was made for each identified emotion. This step involved scrutinizing the text passages within each code, such as motivation. The third step complemented the analysis by identifying the overall themes and the specific emotional geographies.

## Results

Hargreaves’ emotional geography framework was developed because teachers encounter scenarios that can result in positive and negative sociocultural, moral, professional, physical, and political emotions about their relationships with students, parents, colleagues, and the school principal throughout the teaching–learning process ( Chen and Wang, 2011; Hargreaves 2000, 2001a, 2001b; Hargreaves & Lasky 2004; Lassila *et al*., 2017; Liu 2016), which then lead to the development of distinct emotional geographies with other parties. Results of the GPSTs’ interview analyses revealed that the GPSTs had developed emotional distance and closeness from their interactions with the students and cooperating teacher, which had exposed them to all five emotional geographies, the occurrences of which were appended to the respective participant interviews.

### Sociocultural distance: ‘Punctuality is the students’ common problem’

Sociocultural distance typically develops because of differences in cultures, habits, social standing, and economic levels. In this case, all participants expressed negative feelings about the students’ lateness, that is, punctuality was found to be a source of unpleasant emotions (e.g., frustration, anger, and feeling distracted and upset) ( Prasetyarini *et al*., 2021; Bataineh, 2014). The class was able to function normally with few late students; however, class activities were significantly disrupted when over half of the class was late. Most participants stated that they had planned specific tasks for the class, and if the class began late, it affected all activities in the teaching plan, making the participants irritable ( Zaid Bataineh, 2014). Explaining the materials again to the late students, which upset the participants’ concentration, was also necessary, as one participant stated:

Actually, the most emotional part was in the first meeting of teaching practicum; when I started the class, there were around 10 students who attended the class; I asked the leader of the class where her friends were and she said that they were still in the canteen and in other places, and then I asked her to contact her friends because we had to start the class now. For me, yeah, I know it’s like Indonesian habit, yeah, perhaps. Yeah, they were late, and then I had to wait around 15 minutes, and not all of them came to class that day (Participant 1)

This comment reveals that the GPST had experienced unpleasant emotions when the students were late as this had disrupted the lesson plan. However, most GPST participants felt that they had no right to be tough on the students because they were only graduate students, not lecturers ( Boyce, 1997). Therefore, the sociocultural distance associated with student tardiness resulted in the participants experiencing political distance.

### Moral distance: ‘I’m so distracted when they are busy on their phones’

The participants were inevitably confronted with moral distance when they interacted with the students. Moreover, the participants believed that the students occasionally had different moral aims in mind during the interactions. The GPST participants expected the students to listen and pay attention; however, they occasionally encountered students who did not do so, making them feel distracted and enraged, especially when they notice their students were more concerned with their phones, as stated by one participant:

It’s really distracting because we are explaining in front of them and they focus on their phones, and we feel like they aren’t paying attention. We also wonder whether they understood the lesson because we don’t know their feelings about understanding the lesson, and if they were listening or not, so it was really distracting for me (Participant 5)

Another participant added the following:

While I was teaching, I saw that one student was busy on their cell phone. In my heart, I felt so furious, I felt so angry because I’d been preparing my material for 2 hours, but they played with their cell phone, hello! (Participant 14)

Seeing the students ignore them in favor of their phones made them feel humiliated and disrespected ( Baker *et al*., 2012; Smale *et al*., 2021; Thomas & O’Bannon, 2013; Tindell & Bohlander, 2012). They felt that they had tried to do their best when teaching the class but had received disrespectful behavior in return. Inappropriate student behavior has commonly been reported as a source of negative feelings among preservice teachers ( Baker *et al*., 2012; Karunagaran & Saimin, 2019; Thomas & O’Bannon, 2013) as they felt what they were doing was useless because they did not get the responses they wanted.

### Professional distance: ‘Am I competent enough to teach them?’

The most striking conclusion from this study is the emotional distance the participants felt about their professionalism. The GPST participants’ perceptions of their professional worth varied. The first point of contention was when they expressed doubts about their competence to educate the undergraduate students because the students had made them believe that their knowledge was insufficient. In response to this issue, the participants felt anxious during their initial period in the class and when teaching the students. As one participant stated,

I get anxious right after thinking, ‘Will they like me?’ For example, will they accept me as their exchange teacher for the time I am going to teach them, but I am still a master’s degree student, so will they appreciate me? Will they accept me? I get this kind of feelings. (Participant 10)

Not only did the participants cast doubt on their competence but they also acknowledged that they had limited comprehension of the teaching content at times ( Agustiana, 2014). Consequently, the participants felt unable to respond when the students posed critical questions about issues. Another instance was when they believed they had not adequately prepared the content and lacked mastery of the material they had provided to the students. According to one participant, ‘Because there was a topic that I didn’t really understand, I tried to make my face look like I understood it and then I felt like no one noticed’ (Participant 3).

Teachers should be well prepared for class and must comprehend and master the topics they are going to teach. However, preservice students occasionally have difficulties comprehending the content of their teaching materials and often do not have the time to talk with their cooperating teachers. These issues can cause students to have doubts and reservations about the teacher’s competence to instruct in the class and can affect subsequent lessons, even if the teachers are fully prepared afterwards. Therefore, teachers must be professionally prepared to create a favorable educational environment.

Furthermore, the teaching practicums with undergraduate students majoring in English Education required the participants to communicate in English on daily basis. Regrettably, not all participants spoke English fluently and were therefore confronted with a professional divide, as commented on by one participant, ‘Maybe, generally, I have not been really satisfied with my speaking environment in the past years, something like that’ (Participant 13).

The participant expressed dissatisfaction with their speaking ability, which they believed was insufficient ( Daud *et al*., 2019). Owing to a lack of confidence in their ability to communicate in English, they felt incapable of teaching the students. From the students’ perspective, English majors need the lecturer to be proficient in English teaching techniques; therefore, they would not feel they were ideal models for effective English as a Foreign Language (EFL) teachers if the students felt that participants were not competent lecturers. The questioning of a teacher’s ability occurs regularly in Indonesia, primarily because many EFL teachers are non-native English speakers. Thus, students are hesitant to speak English if they are only taught through textbooks; therefore, Indonesian preservice teachers should prioritize the English abilities needed to assist them in their teaching, that is, English fluency is a must for EFL teachers ( Daud *et al*., 2019).

Another difficulty associated with professional distance was when the teacher’s plans did not align with the students’ qualities. Teachers are often confident that certain methods will be successful in the classroom; however, success is contingent upon whether these methods are appropriate for students. Therefore, professional distance occurs when reality does not match the teacher’s expectations, as reported by one participant:

This sometimes happens in our teaching because I had already planned to apply this method; however, when I applied this method and it was not related to the situation in the classroom, I had to directly find another way to match my method and the classroom situation. For example, when I prepared to conduct a group activity, such as a presentation, I saw that only one group was actively joining the presentation, so I had to change and present an activity that could interest all students in the classroom (Participant 5)

The teacher had arranged a group discussion and had anticipated that all students would fully participate; however, the classroom atmosphere or the students’ qualities did not align with the preplanned activities, meaning that the teacher had to choose whether to alter their activities or continue with them regardless of student engagement. When confronted with an unexpected event in the classroom, the decision to change activities needs to be made by the teacher based on their professional judgment, which also means that teachers should have alternative plans when their planned activities do not proceed as expected.

### Physical distance: ‘I was nervous as it was the first time I had taught undergraduate students’

When establishing an emotional bond between teachers and students, there inevitably needs to be an intimacy, regularity, and continuity of engagement between both parties ( Hargreaves, 2001b). Physical closeness and distance are also related to the amount of time or space produced between the parties that interact ( Liu, 2016). The physical theme evolved because of the interactions between the participants and other stakeholders (i.e., the students, cooperating teachers, and administrative staff). Three participants expressed anxiety when confronted with physical distance in their encounters with the students or the cooperating teachers.

The first physical distance occurred when preservice teachers were teaching undergraduate students, with a majority reporting feeling apprehensive when standing in front of students their age ( Boyce, 1997). Their anxiety was because of a sense of inadequacy, unfamiliarity, and distrust. Their reactions to the physical distance were influenced by their first efforts at teaching in front of a class and by the professional distance they felt in the class ( Agustiana, 2014; Shah *et al*., 2021; Zhu, 2017). Their uncertainty about their abilities and characteristics exacerbated their unfamiliarity with the students, which was why they were scared and experienced a physical distance from the students. As one participant stated:

I think the biggest challenge for me was when I tried to explain something to them, given that was my first time teaching undergraduate students. I used to teach kids but not undergraduate students, so it was a big challenge. And then came the anxiety, right after I thought about whether they will like me? (Participant 10)

The aforementioned participant was concerned that the students would reject them because they were only a master’s degree student. This sentiment was shared by the majority of GPST participants in this study who were fearful that the students would question their abilities as they were only novice undergraduate teachers. These difficulties resulted in a range of emotions, including anxiety and nervousness.

Another difficulty related to physical distance occurred during the initial meeting of two participants with their cooperating teachers. Cooperating teachers are the classroom teachers with whom the participants needed to collaborate. Preservice teachers substitute for the cooperating teachers in three to five classes; therefore, maintaining a positive relationship with the cooperating teachers is a requirement so that the preservice teachers can discuss their concerns and seek and discuss solutions. However, not all participants had met their cooperating teachers in person, which created anxiety. As one participant put it:

‘My coordinator was Mrs. A, then I was first felt bit scared because I was not very familiar with my cooperating teacher’ (Participant 7)

The participant encountered physical distance when communicating with their cooperating teacher because they had never met them ( Boyce, 1997). Similar problems were raised by other participants who had never previously communicated with their cooperating teacher. They felt awkward initiating communication with someone they did not know in person as they felt it was necessary to meet their coordinating teacher in person first before discussing the teaching practicum. However, building a personal attachment before the teaching practicum was slightly difficult because not all preservice teachers lived in the same city as the university and their cooperating teacher. Therefore, they tried to contact them using messenger or social media but still felt awkward. This physical distance, in some cases, led to political distance as they felt very nervous when teaching in front of their cooperating teacher.

### Physical closeness: ‘I’d like to be a friend with the students’

Surprising information was found related to physical geography concerning how the preservice teachers felt about their student interactions. The teachers frequently attempted to create distance between themselves and the students to retain their professionalism. However, most of the GPSTs in this study believed that developing a close relationship with the students enabled them to better accomplish their teaching objectives. As one participant stated, ‘I really like teaching and I like to blend with the students’ (Participant 6). Another participant added the following:

I tried to be friends with some of them, although they may feel awkward to befriend me; however, some of them were just like I told you, now I’m now friends with one of the students (Participant 9)

The participants believed that having personal contact with the students could gain their attention and respect; therefore, they attempted to develop positive relationships with the students through laughing, projecting a warm attitude, and providing personal communication. Even after the teaching practicum was completed, one participant stated that they enjoyed becoming friends with the students and developing relationships. The participants did not view their closeness to students to be a violation of their professionalism; rather, they felt that this closeness demonstrated their professionalism. It appeared that the study participants had divergent views on student–teacher interactions.

### Political distance: ‘I’m not even their real lecturer’

The final theme was political geography, which revealed the divergent views about the exercise of power ( Liu, 2016). Political distance was observed when the participants questioned their power or authority in the class or when the teaching practicum coordinators were observing them.

Yeah, it happens. I asked the students an easy question, but there was no answer. I didn’t know if they really had no idea or they just were ignoring me because I was only a practicum teacher and not their real teacher. I was so angry because I felt disrespected. I wanted to go further, but I realized I had no authority for that as I was only a temporary lecturer for them, so I cannot exaggerate. (Participant 15)

This political distance made the GPST participants uneasy, which was also evident in their interactions with their cooperating teachers. They were nervous when they noticed the cooperating teacher, who they knew had greater power and authority, observing them in the class ( Boyce, 1997). In comparison, one participant experienced a sense of political intimacy when their cooperating teacher bestowed complete authority on them and allowed them to teach the class without being monitored.

What I taught in only 15 minutes at the beginning of the class, I could explain clearly and briefly to the students; I could communicate and interact well with the students. But when the teacher entered the class, I suddenly forgot the material. (Participant 13)

Political distance was not found to only occur during the interactions between the participants and their cooperating teacher. Certain situations also made them feel helpless and had a major impact; however, they felt they could not deal with the situation. As one participant put it:

Distractions in the classroom, such as from the students themselves or from the situation in the classroom, when I was conducting my teaching for the first time, the class was very hot because there was no air conditioning, so most students were not focusing and some people outside were being really noisy. (Participant 5)

Therefore, as aforementioned, many GPST participants felt powerless when the classroom environment was not conducive. Classroom settings and the environment can have a substantial impact on class activity ( Marzano & Marzano, 2003). Hot and noisy classrooms can be disruptive to both students and teachers and impair classroom activities ( Prasetyarini *et al*., 2021). Therefore, the participant was unable to intervene because the classroom was hot and excessive noise was noted outside the classroom. The situation prevents students from concentrating on the materials provided by the participant. As a result, she maintains a political distance from the problem.

## Discussion

After examining the negative and positive emotions experienced by the GPSTs when teaching undergraduate students in their teaching practicum, the results were examined more broadly in light of Hargreaves’ (2001b) emotional geography concepts to determine how these emotions related to their teaching paradigms, beliefs, and styles. The GPSTs were confronted with various unfavorable sociocultural, moral, professional, physical, and political emotional barriers throughout their teaching practicums.

The first factor identified was sociocultural distance. Sociocultural distance is generally characterized by a separation of cultures, customs, social standing, and economic status ( Hargreaves, 2001b). The GPST participants encountered a sociocultural distance because of the students’ punctuality, which was similar to the situation encountered by Ragawanti (2015), who discovered that the preservice teachers’ challenges revolved around activity management, with the most common difficulty being activity timing. Unlike students in primary and secondary schools, who typically have a set classroom and exit only at recess, university students spend most of their time outside the classroom waiting for their class to begin, which means a higher possibility exists that the students will arrive late to class. Having students arrive late irritated and angered the GPSTs as the lateness was interpreted as being disrespectful toward their authority as the practicum teacher ( Prasetyarini *et al*., 2021; Zaid Bataineh, 2014). Thus, they questioned their authority and ability to discipline students who were late. Late students also disrupted the participants’ lesson plans and their ability to complete their well-designed exercises within the allocated time ( Zaid Bataineh, 2014). Because many students arrived late to class, they wasted time and the GPSTs were unable to complete their planned tasks.

These late student episodes made the GPSTs feel that teachers should be rigorous when abiding by class regulations and that students should understand the implications of their tardiness and its effect on class activities. Consequently, the GPST participants chose to enforce stringent time limits and advised students that they would be unable to follow the class if they arrived late ( Prasetyarini *et al*., 2021). Therefore, the GPSTs discovered that tight classroom regulations were necessary to ensure student punctuality.

The GPSTs felt moral distance when their aims differed from those of the students. Teachers may encounter student ignorance, excessive noise, silence ( Ragawanti, 2015), and even poor behavior ( Karunagaran & Saimin, 2019). The GPSTs said that they experienced feelings of moral distance when they observe that students’ attention was more focused on their phones than on their lessons, which they felt was disrespectful behavior. They admitted to being enraged, agitated, and preoccupied with the situation and believed that even though they had planned and prepared everything necessary to teach the students, the students lacked respect for them as teachers.

This moral distance encouraged the GPSTs to perceive the situation from both their and the students’ perspectives because they wanted to understand why the students disregarded them and preferred to play games on their phones than listen to the lesson ( Baker *et al*., 2012; Smale *et al*., 2021; Thomas & O’Bannon, 2013; Tindell & Bohlander, 2012). Many of the GPSTs believed that it was either their teaching style that was boring the students or that their prepared activities were not suitably engaging. These beliefs compelled the preservice student teachers to admit that they needed to adapt their teaching approaches to make them more appropriate and appealing to the students in order to avoid boredom ( Karunagaran & Saimin, 2019).

Professional geographies are formed when professional definitions and norms either divide the involved parties or allow them to collaborate on professional concerns. The GPSTs reported feeling professional distance in their relationships with their students and experienced certain emotions because they (1) had doubts about their abilities, (2) had not mastered the content, and (3) could not communicate fluently in English. The first difficulty arose when they mistrusted their abilities to teach the students. Most GPSTs in this study expressed similar concerns about the student’s perceptions of their abilities and were fearful that if they could not teach well, they would be ignored ( Daud *et al*., 2019) and that their knowledge was insufficient to facilitate learning, all of which gave rise to anxiety, worry, and nervousness when teaching.

The second difficulty was because many of the GPSTs struggled to master the subject matter they were assigned to teach, and those who were confronted with this issue acknowledged that they lacked a thorough understanding of both the subject and the materials. While most attempted to comprehend the material, many were unable to do so but pretended to know it to avoid answering student inquiries ( Agustiana, 2014). This was a legitimate concern regarding their professionalism. Teachers should be confident about their materials and activities before entering the class as this confidence can make it easier to control the class. Therefore, neglecting to master the content could foster a dislike toward the teacher. The GPSTs in this study acknowledged that they required additional time to become acquainted with the materials; however, most claimed that they had had only a limited amount of time to prepare and were therefore unable to do their best. They also assumed that learning the content would help them control their negative emotions in front of the students.

Finally, the participants expressed dissatisfaction with their speaking abilities as a result of the professional distance ( Daud *et al*., 2019). As EFL teachers, they were obligated to communicate in English; however, not all GPSTs in this study could communicate fluently in English, making them feel embarrassed and dissatisfied with their competence, primarily because most believed that English major students should have excellent teachers that can serve as EFL teacher role models. Most participants had shattered expectations because of their inability to communicate fluently with the students, enabling them to be aware of the necessity to practice their speaking in order to improve their teaching effectiveness as EFL teachers.

Physical geographies are concerned with time and place and connect or reduce relationships ( Hargreaves, 2001b). The GPSTs encountered both physical distance and closeness in their interactions with the students and the cooperating teacher. They reported feeling apprehensive when teaching for the first time ( Agustiana, 2014; Shah *et al*., 2021; Zhu, 2017), with their anxiety being exacerbated by their doubts about their ability to instruct the undergraduate students. A similar feeling occurred when they were required to communicate with their cooperating teachers for the first time. Several participants acknowledged that they had never met their cooperating teachers in person, which made contacting them initially awkward; however, this physical distance occurred only during the earliest stages of the relationship as they became calmer and more capable of reducing the physical gap when they had become better acquainted with the students and the cooperating teachers.

The participants perceived physical closeness during their teacher–student interactions despite their physical distance from the students. Most GPST participants believed that becoming better connected to the students as friends could assist them in maintaining class control. However, these beliefs contradict an earlier study that found that teachers preferred to maintain a professional distance between themselves and their students to remain professional ( Liu, 2016). However, the GPSTs in this study defined professionalism differently than typical teachers, believing that being personally appealing and easy to g *et al*ong with would be of benefit in their future careers. They felt that they needed to treat the undergraduate students differently because these students were adults who, unlike children and adolescents, could not be managed only by punishments or rewards. Given these disparate features, the GPST participants concluded that they needed to become friends with the students to enjoy the class and ensure success with the class activities.

The final theme was political geography, which is related to divergent views of power ( Liu, 2016). The GPSTs reported experiencing unpleasant emotions when confronted with certain concerns (e.g., their authority in class, being observed by the cooperating teachers, and their inability to change unfavorable circumstances). The first political distance issue was inextricably linked to moral distance when the participants experienced negative feelings from the students’ behavior and powerlessness to intervene because they believed they lacked authority. Another concern was when the cooperating teachers were observing the participants ( Agustiana, 2014; Boyce, 1997), with most GPSTs admitting that being monitored made them feel restricted because they were fearful of making errors ( Daud *et al*., 2019). Several participants stated that they had been trembling when the cooperating teachers were observing them teach. However, most also felt that the observations made by the cooperating teachers’ could assist them. The final point was about the sense of impotence the GPSTs felt when confronted with unfavorable circumstances, the participants in this study complaint about the external factors like uncomfortable classroom which lead them to feel powerless since they have no authority or power to solve the problems. 

### Limitation and implication

Regarding its limitations, the researchers identified a number of areas for improvement, including the sample method and the interview procedure. The participants in this study were taken randomly from one class of master's degree students who had finished their teaching practicum. Therefore, the researchers ware unable to characterize greater variance in the emotions displayed by the participants, as they were homogeneous. Consequently, the current study recommends that future research include a prior study through a survey and supplement it with an interview in which the researchers may determine which features are of interest. In addition, since the present research was conducted in the context of the COVID-19 pandemic, where the researchers were unable to meet the participants in person and conducted the interview via Zoom, the researchers encountered several difficulties, such as poor internet connectivity, which led to misunderstandings between the interviewer and the interviewee regarding the topic discussed; consequently, the answers of several participants cannot be utilized for further analysis. On the basis of this constraint, the researchers suggest that future studies conduct face-to-face interviews with the participants in order to clarify any misconceptions immediately. Furthermore, since this study relied on pre-service teachers' experienced during the teaching practicums which emphasized on their feelings only, it will be a better enrichment when the future research can correlate their experience with the way they cope with it, so called their self-regulation **.**

## Conclusion

This study demonstrated the validity of using emotional geographies to describe the beliefs, paradigms, and styles of preservice student teachers. Hargreaves’s (2001a, 2001b, 2004) emotional geographies were applied to broaden the scope of the recorded examples. While the emotional geography concept has been previously applied to education, it has rarely been applied in preservice research. The current study demonstrated that the emotional geography concept could assist researchers to map preservice student teachers’ views during their practicums.

The teaching practicums with undergraduate students engendered some negative emotions, resulting in emotional distance. However, participants claimed that they could use these unpleasant emotions to improve their teaching styles and beliefs, concluding that teachers needed to maintain class discipline. Most also believed that teachers needed to present materials appealingly to avoid student boredom, that teachers should make friends with the students to maintain control in the classroom, and that thorough preparation was necessary to instill confidence in the students and be able to respond to their questions. Overall, however, both the positive and negative emotions experienced during their teaching practicum with the undergraduate students provided the GPST participants with significant experiences that will benefit them in their future careers.

## Data Availability

Figshare: Interview Transcript of Graduate Student Teachers, https://doi.org/10.6084/m9.figshare.22276717.v2 ( Sunardi *et al*., 2023a). This project contains the following underlying data:
•
Interview with Graduate Student (Participant 1).docx
•
Interview with Graduate Student (Participant 2).docx
•
Interview with Graduate Student (Participant 3).docx
•
Interview with Graduate Student (Participant 4).docx
•
Interview with Graduate Student (Participant 5).docx
•
Interview with Graduate Student (Participant 6).docx
•
Interview with Graduate Student (Participant 7).docx
•
Interview with Graduate Student (Participant 8).docx
•
Interview with Graduate Student (Participant 9).docx
•
Interview with Graduate Student (Participant 10).docx
•
Interview with Graduate Student (Participant 11).docx
•
Interview with Graduate Student (Participant 12).docx
•
Interview with Graduate Student (Participant 13).docx
•
Interview with Graduate Student (Participant 14).docx
•
Interview with Graduate Student (Participant 15).docx Figshare: Analyzed Interview Transcript, https://doi.org/10.6084/m9.figshare.22191286.v2 ( Sunardi *et al*., 2023b). This project contains the following extended data:
• Emotional Geographies fix.xlsx Data are available under the terms of the Creative Commons Attribution 4.0 International license (CC-BY 4.0).
