# Supplementary material for: A phenomenological analysis of the emotional experiences of graduate preservice student teachers in Indonesia
Source: F1000Res. 2025 May 6;12:505. Originally published 2023 May 16. [Version 3] doi: 10.12688/f1000research.132112.3 (PMC12082072; doi:10.12688/f1000research.132112.3)
Supplement: Peer review history

# Reviewer report: Reviewer response for version 3

**DOI:** 10.5256/f1000research.181229.r383098

**Published:** 2025-05-13

**Authors:** Xuesong Gao

**Affiliations:** 1 The University of New South Wales, New South Wales, Australia

I have no further issue with the study.

Is the work clearly and accurately presented and does it cite the current literature?

Yes

If applicable, is the statistical analysis and its interpretation appropriate?

Not applicable

Are all the source data underlying the results available to ensure full reproducibility?

Yes

Is the study design appropriate and is the work technically sound?

Yes

Are the conclusions drawn adequately supported by the results?

Yes

Are sufficient details of methods and analysis provided to allow replication by others?

Partly

Reviewer Expertise:

language teacher education

I confirm that I have read this submission and believe that I have an appropriate level of expertise to confirm that it is of an acceptable scientific standard.

# Reviewer report: Reviewer response for version 2

**DOI:** 10.5256/f1000research.178867.r378436

**Published:** 2025-04-24

**Authors:** Xuesong Gao

**Affiliations:** 1 The University of New South Wales, New South Wales, Australia

I suggest that the authors include research questions that were addressed in the study. Research question(s) should be well motivated by the literature review (and introduction) and presented separated from the rest of writing to be more visible. 

 In the methodology section, it should be made clear how the data were collected and analyzed to answer the research question(s). 

 Without reference to the research question(s), it is difficult for this reader to appreciate whether the study has achieved what it was supposed to achieve.

Is the work clearly and accurately presented and does it cite the current literature?

Yes

If applicable, is the statistical analysis and its interpretation appropriate?

Not applicable

Are all the source data underlying the results available to ensure full reproducibility?

Yes

Is the study design appropriate and is the work technically sound?

Yes

Are the conclusions drawn adequately supported by the results?

Yes

Are sufficient details of methods and analysis provided to allow replication by others?

Partly

Reviewer Expertise:

language teacher education

I confirm that I have read this submission and believe that I have an appropriate level of expertise to confirm that it is of an acceptable scientific standard, however I have significant reservations, as outlined above.

# Response

**Published:** 2025-04-28

**Authors:** Famala Eka Sanhadi Rahayu

**Affiliations:** Universitas Mulawarman, Indonesia

Dear Reviewer

 We sincerely thank the reviewer for the insightful and constructive feedback provided. In response to the comments, we have carefully revised the manuscript to enhance its clarity, structure, and alignment with the study's objectives. The revisions undertaken are as follows:

**1. Addition of Explicit Research Questions (Separated and Clearly Visible)**

 To address the reviewer's suggestion, we have inserted a new section titled **“Research Questions”** immediately following the Introduction and preceding the Literature Review. This section explicitly states the two research questions that guide the study.

**Revision Inserted:**

**Research Questions**

 This study is guided by the following research questions:

1. *What types of emotional geographies (sociocultural, moral, professional, physical, and political) are experienced by Graduate Preservice Student Teachers (GPSTs) during their teaching practicum?*
2. *How do these emotional experiences influence GPSTs' teaching beliefs, teaching styles, and professional development during their practicum?*

**2. Stronger Motivation of Research Questions from the Introduction**

 We have refined the concluding paragraphs of the Introduction to more clearly highlight the research gap and to lead logically into the formulation of the research questions.

**Revision Inserted at the End of Introduction:**

*Although prior studies have examined teachers’ emotional experiences with parents (Chen & Wang, 2011; Sanhadi Rahayu & Asanti, 2021), students (Hargreaves, 2000), colleagues (Hargreaves, 2001a), and principals (Lassila et al., 2017), little attention has been given to preservice teachers’ emotional geography during practicums. Thus, the current study addresses this gap.*

**3. Clarification of Data Collection and Analysis in Relation to the Research Questions**

 To enhance clarity, we have explicitly detailed in the Methods section how the data collection and analysis procedures were designed to address the research questions.

**Revision Inserted into Instruments Subsection:**

*This study used semi-structured interviews based on Hargreaves' five emotional geography dimensions. The interview structure was designed specifically to explore the emotional geographies (RQ1) and their impact on teaching beliefs and styles (RQ2).*

**Revision Inserted into Research Procedure Subsection:**

*These interviews were designed to gather in-depth qualitative data needed to answer the stated research questions regarding types of emotional geographies experienced and their influence on teaching beliefs and practices.*

**Revision Inserted into Data Analysis Technique Subsection:**

*A phenomenological approach was adopted because it best supports exploring participants' lived experiences related to emotional geographies, directly aligning with the research questions.*

*The data analysis systematically addressed the types of emotional geographies encountered (RQ1) and their influence on GPSTs’ professional development (RQ2).*

 We hope that these revisions have sufficiently addressed the reviewer’s concerns and improved the overall clarity and rigor of the manuscript. All changes have been clearly marked in the revised manuscript for ease of review.

# Reviewer report: Reviewer response for version 2

**DOI:** 10.5256/f1000research.178867.r377400

**Published:** 2025-04-15

**Authors:** Maria Olímpia Paiva

**Affiliations:** 1 University of Minho, Braga,, Portugal

I would like to commend the authors for the revisions made to the article. The updated version provides greater clarity regarding the study’s objectives and methodology. The theoretical foundations that were previously ambiguous have been sufficiently clarified. These changes enhance the rigour and readability of the work. I believe the authors have adequately addressed the concerns raised in the initial review. 

 I have no further comments at this time.

Is the work clearly and accurately presented and does it cite the current literature?

Yes

If applicable, is the statistical analysis and its interpretation appropriate?

Not applicable

Are all the source data underlying the results available to ensure full reproducibility?

Yes

Is the study design appropriate and is the work technically sound?

Partly

Are the conclusions drawn adequately supported by the results?

Yes

Are sufficient details of methods and analysis provided to allow replication by others?

Partly

Reviewer Expertise:

.

I confirm that I have read this submission and believe that I have an appropriate level of expertise to confirm that it is of an acceptable scientific standard.

# Reviewer report: Reviewer response for version 1

**DOI:** 10.5256/f1000research.145015.r299520

**Published:** 2024-07-17

**Authors:** Maria Olímpia Paiva

**Affiliations:** 1 University of Minho, Braga,, Portugal

Dear authors,

 Here are some suggestions to make the article clearer, more objective, and of excellence:

 Abstract

 The objectives of the study should be more explicitly stated in the summary, as they are currently diluted between the "Context" and "Method" sections, and there should be a specific heading – "Objectives".

 Introduction

 - Rewrite the underlined text to avoid repeating "Hargreaves, 2001b" too often:

*“Emotional geographies describe the patterns of closeness and distance in human*

*interactions that shape the emotions we experience about relationships to ourselves, each other, and the world around us (Hargreaves, 2001b). Hargreaves (2001b) claimed that understanding teachers ’ emotions is crucial because it can influence the classroom environment and the conduct of the learning activities. Hargreaves ’ (2001b) emotional geography framework can also be used to map a teacher ’ s series of feelings. Hargreaves (2001b) defined five emotional geographies (i.e., sociocultural, moral, professional, physical, and political) as geographical and experiential patterns of closeness/distance in human connections that form, configure, and color feelings about ourselves, the world, and one another.”*

 - As the article is based on Hargreaves' (2001) theoretical model of emotional geographies, it is essential that this model is further developed, along with its dimensions (socio-cultural, moral, professional, physical, and political). The author considers that teaching and learning are undoubtedly emotional practices, and consequently, the school space becomes an emotional geography. Hargreaves argues that such constructivist approaches to learning place greater emphasis on problem-solving and the application of knowledge, broadening the focus on creativity. In this context, emotional geographies describe the patterns of closeness and distance in human interactions, shaping the emotions we experience in our relationships with ourselves, others, and the world around us. Thus, emotion and cognition, feeling and thinking, intertwine in all social practices in complex ways.

*“However, the investigation of emotional geography of preservice teacher during the*

*practicum has remained unseen by many scholars. Therefore, the present researchers want to fill this gap through this study”*

 - First and third person references should be avoided. The information mentioned above should be addressed in the study objectives.

*“They have to deal with various settings involving students and the sociocultural contexts in the institution in which they are teaching (Riesky, 2013). This study revealed the difficulties preservice teachers experienced due to their emotional beliefs (Riesky, 2013).”*

 Rewrite the underlined text to avoid repeating "Riesky, 2013”.

*“A teacher’s experience of teaching can be affected by their relationships with the students (Hargreaves, 2000), parents (Hargreaves & Lasky, 2004), their colleagues (Hargreaves, 2001a), principals, and educational changes (Hargreaves, 1998a, 1998b), with the feelings from these relationships affecting teaching styles, judgments, and the conduct of the class learning activities.”*

 Other authors' studies can be referenced in addition to those associated with Hargreaves.

 Before presenting the empirical part of the study, it is suggested to briefly outline the study objectives.

 Methods

 The methodology should be guided by the headings "Participants," "Procedures," "Instruments," and "Data Analysis," under which all relevant information pertaining to each heading should be included.

 Ethics

 This part concerning "Ethics" should be included under "Procedures."

 Participants

 Characterize the sample more comprehensively by including gender, years of service, age, and whether they work in public or private education, …

 Research procedure

 The information in Table 1 is not easy to understand.

 Dats analysis technique

 In the "Data Analysis" section, the "Instrument" used consists of the three mentioned stages (emotion coding, identification and/or interpretation of causes of reported emotions, and identification of themes based on emotional geography structure), based on Hargreaves (1998a). However, it is unclear how these three stages were adapted to the Indonesian context and what procedures were taken to validate these stages. The entire validation process and reliability of these three stages must be clarified to validate the study results effectively.

 Limitation and Implication

 Due to the known challenges in qualitative studies, it is suggested to propose additional considerations for future research to address the identified limitations, providing a more comprehensive and profound understanding of the emotional experiences of novice teachers and contributing to continuous improvement in teacher education. Examples to consider include: inclusion of various educational contexts; socio-cultural diversity; mixed data collection methodologies; longitudinal studies; enhanced communication tools; automatic recording and transcription; emotion analysis using AI tools; study of self-regulation strategies; assessment of pandemic impacts; hybrid teaching models; emotional support programs; and training in emotional self-regulation.

 Conclusion

 Considering the importance of the topic, in addition to the conclusions presented, other relevant aspects for the training of future teachers can be considered. By incorporating these aspects into the training of future teachers, it is possible to prepare them in a more holistic and effective manner to face the challenges of contemporary education, promoting quality and student-centered teaching. Some suggested examples include: Development of Socioemotional Skills; Practices of Self-reflection; Integration of Educational Technologies; Continuous Professional Development; Diversity and Inclusion; Innovative Pedagogical Strategies; Collaboration and Teamwork.

Is the work clearly and accurately presented and does it cite the current literature?

Yes

If applicable, is the statistical analysis and its interpretation appropriate?

Not applicable

Are all the source data underlying the results available to ensure full reproducibility?

Yes

Is the study design appropriate and is the work technically sound?

Partly

Are the conclusions drawn adequately supported by the results?

Yes

Are sufficient details of methods and analysis provided to allow replication by others?

Partly

Reviewer Expertise:

.

I confirm that I have read this submission and believe that I have an appropriate level of expertise to confirm that it is of an acceptable scientific standard, however I have significant reservations, as outlined above.

# Response

**Published:** 2024-07-18

**Authors:** Famala Eka Sanhadi Rahayu

**Affiliations:** Universitas Mulawarman, Indonesia

Thank you very much for your suggestions. I will definitely make an improvement based on what you suggested.
